# Sex-related pharmacokinetic differences and mechanisms of metapristone (RU486 metabolite)

**DOI:** 10.1038/s41598-017-17225-0

**Published:** 2017-12-07

**Authors:** Wenge Chen, Yingying Xiao, Jianzhong Chen, Jian Liu, Jingwei Shao, Tao Li, Yewei Zhu, Ji Ma, Yu Gao, Jichuang Wang, Jianguo Xu, Yusheng Lu, Lee Jia

**Affiliations:** 1 Cancer Metastasis Alert and Prevention Center, and Biopharmaceutical Photocatalysis, State Key Laboratory of Photocatalysis on Energy and Environment, Fuzhou University, Fuzhou, 350002 China; 2 Fujian Provincial Key Laboratory of Cancer Metastasis Chemoprevention, Fuzhou University, Fuzhou, 350002 China; 3 Sunlight Building, 6FL; Science Park, Xueyuan Road, University Town; Cancer Metastasis Alert and Prevention Center, Fuzhou University, Fuzhou, Fujian 350002 China

## Abstract

Metapristone is the primary metabolite of the abortifacient mifepristone (RU486), and is being developed as a safe and effective cancer metastatic chemopreventive agent for both sexes. Here, we systematically investigated the sex-related pharmacokinetics of metapristone in both rats and dogs, and explored the related mechanisms of actions. Administration of metapristone to rats and dogs showed that plasma concentrations of metapristone (*AUC*, *C*_*max*_) were significantly higher in female dogs and rats than in males. The sex-related differences in pharmacokinetics become more significant after ten consecutive days of oral administration. Female liver microsomes metabolized metapristone significantly slower than the male ones. The results from P450 reaction phenotyping using recombinant cDNA-expressed human CYPs in conjunction with specific CYP inhibitors suggested that CYP1A2 and CYP3A4 are the predominant CYPs involved in the metapristone metabolism, which were further confirmed by the enhanced protein levels of CYP1A2 and CYP3A4 induced by 1-week oral administration of metapristone to rats. The highest tissue concentration of metapristone was found in the liver. The study demonstrates, for the first time, the sex-related pharmacokinetics of metapristone, and reveals that activities of liver microsomal CYP1A2 and CYP3A4 as well as the renal clearance are primarily responsible for the sex-related pharmacokinetics.

## Introduction

In our effort to identify safe and effective cancer metastasis chemopreventives for asymptomatic cancer survivors, we thought metapristone (the major active metabolite of mifepristone) might be a good candidate for cancer metastatic chemoprevention because of its biostability and few side effects. We hence modified the chemical structure of mifepristone, and synthesized N-monodemethyl mifepristone (metapristone) in a large-scale, and fully characterized the physicochemical properties and bioactivities of metapristone^1^. Metapristone showed a cytostatic effect on cancer cells, and arrested cancer cells at G0 phase and apoptosized the cell. Metapristone interrupted adhesion of HT-29 cells to endothelial cells. Pharmacoproteomic analysis using iTRAQ technique revealed that metapristone intervened with the epithelial marker E-cadherin and mesenchymal marker vimentin in breast cancer cells, and this result constructed its basis for cancer metastasis chemoprevention^2^.

Mifepristone and metapristone share many pharmacological similarities: they interfere with adhesion of cancer cells to the intima of microvasculature by down-regulating cellular expression of integrins^3^ and cellular adhesion molecules^4^, resulting in metastatic chemopreventive effects on mouse models^5^.

In order to develop these abortifacients into cancer metastatic chemopreventives, we further investigated or revisited the safety profiles of mifepristone and metapristone^3,6^. Thirty-day oral administration of mifepristone/metapristone to rats (12.5, 50 and 200 mg/kg/day) caused reversible hepatotoxicity that only occurred at 200 mg/kg/day group as evidenced by the elevated liver enzyme activity and liver organ weight. The long-term toxicity study suggests the potential hepatotoxicity produced by mifepristone or metapristone after their long-term administration at a high dose, but not by their single low doses for abortion.

In the early development of metapristone into a cancer metastatic chemopreventive, we established a robust UPLC/MS/MS-based analytic method to determine blood concentrations of metapristone^7^. In the course of analyzing rat blood concentrations of metapristone, we accidentally found that blood levels of metapristone in female rats were higher than in male rats, suggesting that pharmacokinetic profile of metapristone may be sex-dependent, which may affect the drug’s safety and efficacy. This finding has not been reported before. We therefore conducted the present study to systematically determine the sex-related pharmacokinetics of metapristone in rats and dogs following its administration, and explore the related mechanisms, as well as the drug’s tissue disposition that may relate to its liver toxicity as shown at high doses.

## Results

### Sex-related pharmacokinetics of metapristone

**Figure 1:**
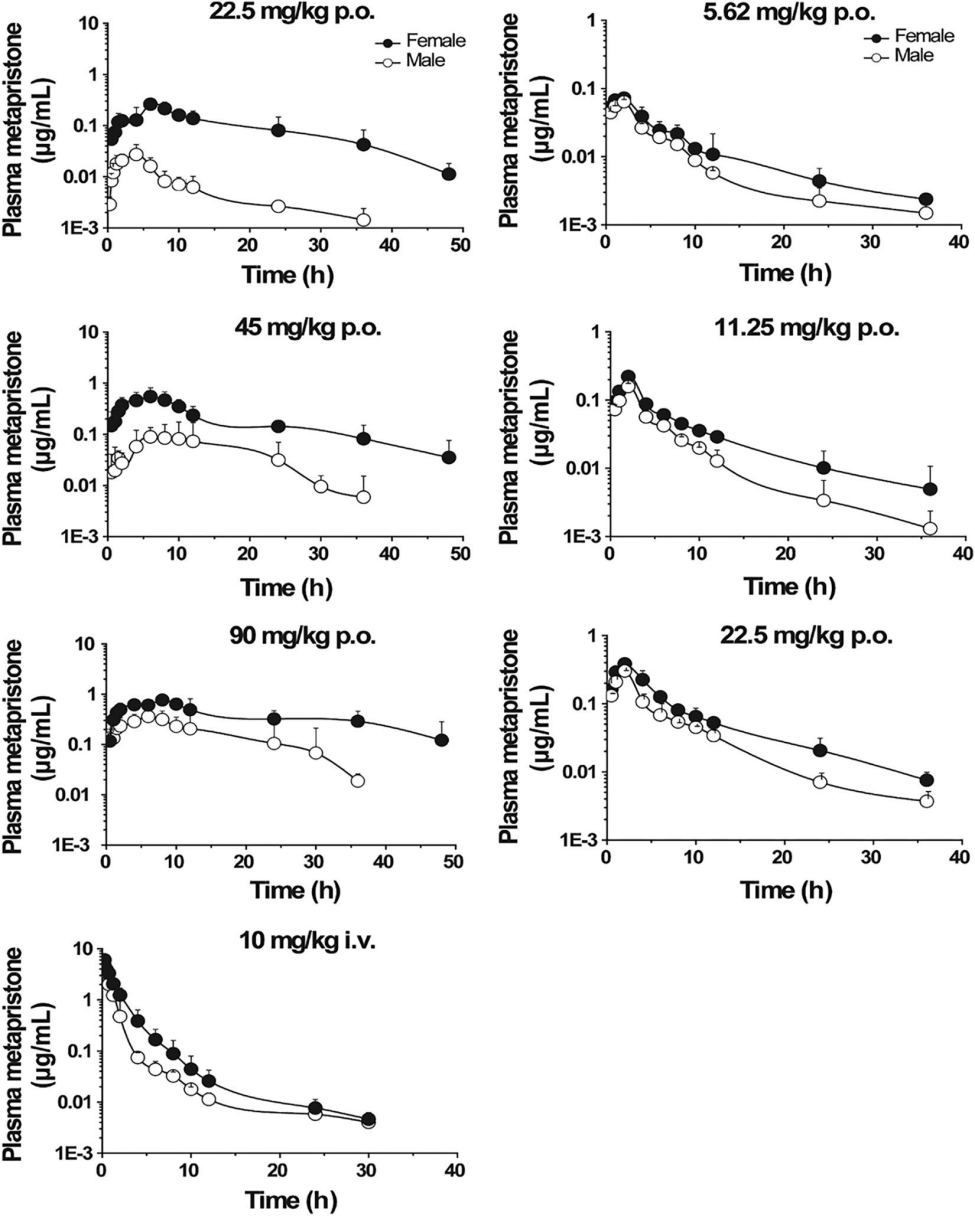
Plasma concentration-time courses of metapristone released after p.o. or i.v. administrations to male and female rats (left) and beagle dogs (right). Each point represents the mean ± s.d. of six rats or three dogs. Note, significant differences between sexes existed in areas under plasma concentrations of metapristone based on the ANOVA analysis.

The mean plasma concentration–time curves of metapristone after single oral or intravenous administration to male and female rats are illustrated in Fig. 1 (left panel). As shown in Table 1, metapristone, after oral absorption, reached its *T*_*max*_ in 5.7–11 h. The *C*_*max*_ of metapristone in females was significantly higher than that in males as shown in Table 1 that was analyzed by using the non-compartmental pharmacokinetic model (Table 1). For instance, at *T*_*max*_, the *C*_*max*_ was 0.14 ± 0.10 and 0.65 ± 0.22 mg/L for male and female rats, respectively, receiving oral metapristone of 45 mg/kg (a magnitude of 364% increase for female rats); the C_max_ was 0.05 ± 0.00 and 0.07 ± 0.01 mg/L for male and female dogs, respectively, receiving oral metapristone of 5.6 mg/kg (a magnitude of 40% increase for female dogs). The differences between female and male rats in *CL/F*, *V*_*d*_ */F* and *AUC*(_*0−t*)_ of metapristone were significant (*P* < 0.05) after intravenous or oral administration of metapristone. The clearance rate *CL/F* in female rats was significantly slower than in male rats. For example, the *CL/F* was 46.9 ± 20.7 and 5.25 ± 0.96 L/h/kg for male and female rats, respectively, receiving oral metapristone of 45 mg/kg (a magnitude of 793% decrease for female rats); the *CL/F* was 1.98 ± 0.39 and 1.06 ± 0.25 L/h/kg for male and female rats, respectively, receiving intravenous metapristone of 10 mg/kg (a magnitude of 87% decrease for female rats). The slow clearance rate of metapristone in female rats may contribute to the significant large *AUC* values found in female rats receiving either p.o. or i.v. metapristone. Oral bioavailability of metapristone at three doses was higher in female rats (ranging from 16.9–18.5%) than in male rats (ranging from 2.1–17.6%; Table 1). The relationship between a single oral dose 22.5, 45 or 90 mg/kg of metapristone and the corresponding area under the plasma concentration-time curves was linear in female rats (Table 1, Fig. 1 left panel).

**Table 1:** Pharmacokinetic parameters of metapristone in rats.

Parameters	p.o.22.5 mg/kg		p.o. 45 mg/kg		p.o. 90 mg/kg		i.v. 10 mg/kg	
	Male	Female	Male	Female	Male	Female	Male	Female
*C*_*max*_ (mg/L)	0.03 ± 0.01	0.27 ± 0.05*	0.14 ± 0.10	0.65 ± 0.22*	0.55 ± 0.18	0.79 ± 0.20*	4.05 ± 0.25	6.05 ± 0.67*
*T*_*max*_ (h)	5.7 ± 2.0	9.0 ± 7.5	7.7 ± 2.9	11.0 ± 12.3	10.3 ± 2.7	7.58 ± 3.38	—	—
*t*_*1/2*_ (h)	8.0 ± 2.4	10.81 ± 3.2	8.11 ± 4.31	10.71 ± 3.71	4.62 ± 3.00	8.83 ± 7.30	9.45 ± 4.993	5.18 ± 3.41
*MRT*_(*0-t*)_ (h)	11.2 ± 2.12	14.42 ± 5.65	13.91 ± 1.17	13.73 ± 6.91	12.75 ± 2.43	15.62 ± 5.82	1.56 ± 0.41	1.68 ± 0.72
*AUC*_(*0*-*t*)_ (mg/L*h)	3.57 ± 1.09	4.05 ± 1.62*	1.96 ± 1.32	9.63 ± 4.54*	8.76 ± 0.07	14.86 ± 2.19*	5.52 ± 1.15	9.75 ± 2.32*
*AUC*_(*0-∞*)_ (mg/L*h)	4.47 ± 1.11	4.23 ± 1.64	2.04 ± 1.33	11.31 ± 3.17*	8.92 ± 0.96	15.56 ± 2.10*	5.46 ± 1.15	9.81 ± 2.34*
*V*_d_ */F* (L/kg)	803.5 ± 266.9	77.32 ± 36.0*	768.0 ± 126.6	67.63 ± 47.6*	92.5 ± 91.7	86.33 ± 62.9*	24.34 ± 5.52	8.02 ± 6.13*
*CL/F* (L/h/kg)	69.61 ± 14.29	7.03 ± 5.50**	46.93 ± 20.7	5.25 ± 0.96**	11.74 ± 3.02	4.85 ± 1.76*	1.98 ± 0.39	1.06 ± 0.25*
*F* (%)	28.7	18.46	7.90	22.02	17.63	16.87	—	—

*P < 0.05, **P < 0.01, compared with those of males.

The mean plasma concentration–time profiles following single oral administration of metapristone to beagle dogs are shown in Fig. 1 (right panel). The relevant non-compartmental parameters are listed in Table 2. At 5.62, 11.25 and 22.5 mg/kg doses, *C*_*max*_ and *AUC* of metapristone in female dogs were about 1–2.4 fold greater than the corresponding *C*_*max*_ and *AUC* in male dogs. On the contrary, the values of *CL/F* in female dogs were 1.5–3.6 fold lower than in male dogs. The values of *V*_*d*_ */F* were 1.3–2.38 fold lower in female dogs than in male dogs. *AUC* increased in a dose-dependent manner in the dogs, and significant differences were found in the main dog pharmacokinetic parameters among the three dose groups. The observations and findings in the dogs were in agreement with what were found in the rats.

**Table 2:** Pharmacokinetic parameters of oral metapristone in beagle dogs.

Parameters	p.o. 5.6 mg/kg		p.o. 11.25 mg/kg		p.o. 22.5 mg/kg	
	Male	Female	Male	Female	Male	Female
*C*_*max*_ (mg/L)	0.05 ± 0.00	0.07 ± 0.01*	0.15 ± 0.03	0.19 ± 0.03*	0.30 ± 0.02	0.38 ± 0.01*
*T*_*max*_ (h)	2.00 ± 0.00	1.67 ± 0.57	2.00 ± 0.00	1.67 ± 0.57	2.00 ± 0.00	1.66 ± 0.57
*t*_*1/2*_ (h)	8.18 ± 2.90	8.05 ± 1.12	5.54 ± 2.30	8.57 ± 1.12	6.79 ± 2.81	7.19 ± 2.83
*MRT*_(*0-t*)_ (h)	7.16 ± 1.12	7.23 ± 1.69	5.68 ± 1.10	8.62 ± 0.56	6.18 ± 0.34	7.00 ± 0.61
*AUC*_(*0-t*)_ (mg/L*h)	0.21 ± 0.03	0.56 ± 0.11*	0.75 ± 0.10	1.28 ± 0.10*	1.56 ± 0.31	2.40 ± 0.15*
*AUC*_(*0-∞*)_ (mg/L*h)	0.23 ± 0.05	0.58 ± 0.12*	0.77 ± 0.11	1.33 ± 0.11*	1.60 ± 0.34	2.56 ± 0.21*
*V*_*d/F*_ (L/kg)	187.10 ± 8.21	125.60 ± 7.10*	136.30 ± 13.08	104.80 ± 9.17*	123.10 ± 15.00	89.23 ± 14.10*
*CL*_*/F*_ (L/h/kg)	15.93 ± 1.60	9.92 ± 0.38*	14.48 ± 1.30	8.48 ± 0.18*	12.59 ± 2.19	8.83 ± 0.76*

*P < 0.05, compared with those of males.

**Figure 2:**
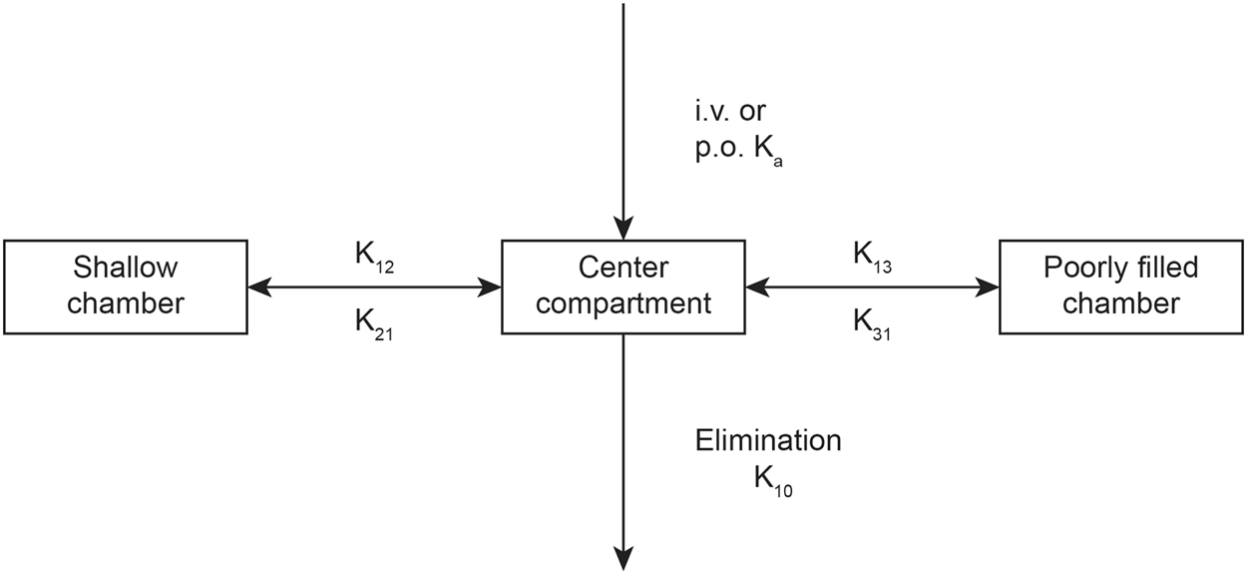
The three-compartmental model of metapristone released once absorbed. The model is composed of the central compartment, shallow chamber, and poorly-filled chamber. Metapristone, after absorbed into the circulation system via i.v. or p.o. administrations, is distributed from the central compartment into either shallow chamber with the fast constant rate (K_12_), or poorly-filled chamber with the slow constant rate (K_13_). Blood metapristone travels back to the central compartment with the constant rates K_21_, or K_31_.

To establish the pharmacokinetic compartment model for metapristone, we used the DAS 3.2 software to model the absorption, distribution and elimination rulings of metapristone. The analysis showed that metapristone in rats and dogs fit, in general, into a three-compartment model that is composed of 1) the central compartment (the main blood stream), 2) shallow chamber (organs and tissues that are well-filled with blood, such as heart, liver), and 3) blood poorly-filled chamber (such as fat and skin). Figure 2 illustrated the model.

**Figure 3:**
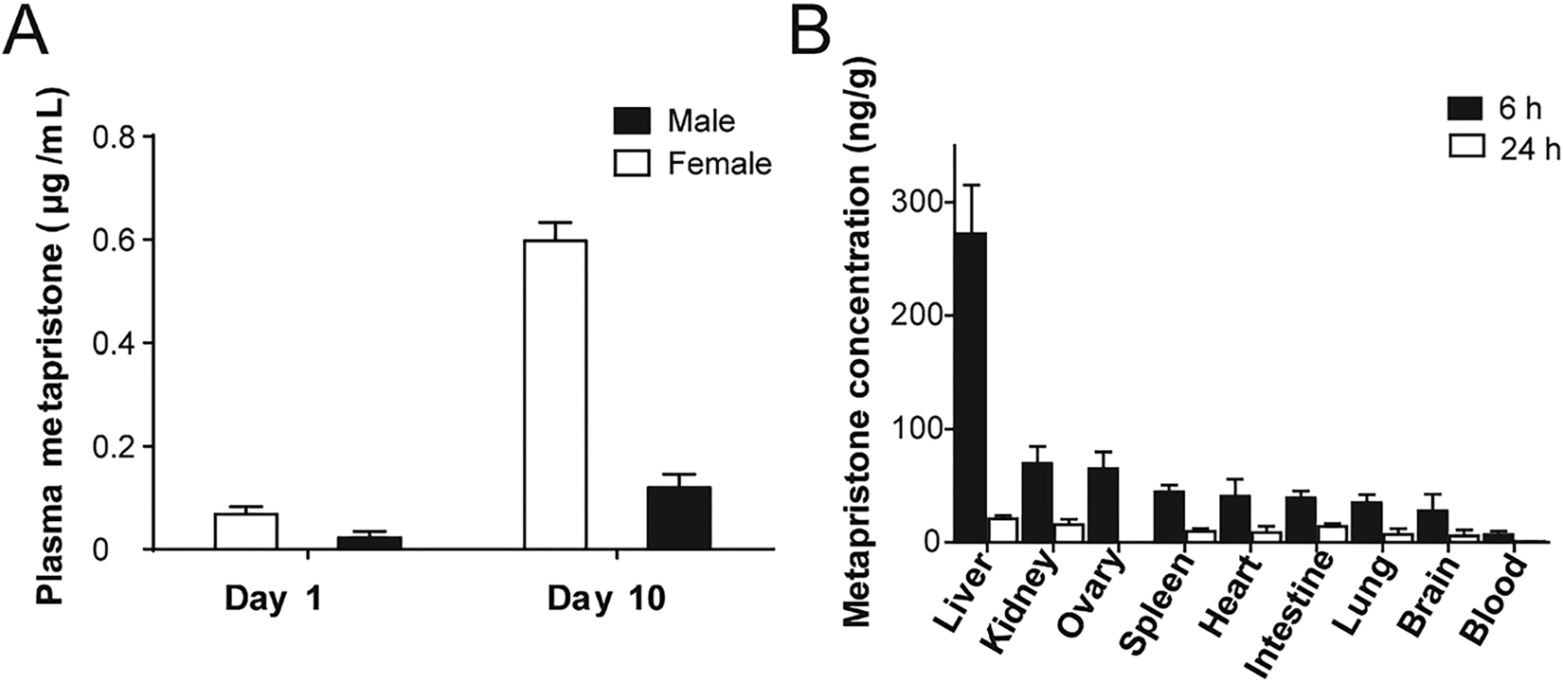
Plasma concentrations and tissue levels of metapristone released. (**A**) Accumulated metapristone in blood at 6 h following 10 consecutive days of oral administration of metapristone to rats. Plasma metapristone in females was significantly higher than in males. (**B**) Female rat tissue concentrations of metapristone at 6 and 24 h after single oral administration. Each bar represents the mean ± s.d. of 4 rats receiving 45 mg/kg metapristone.

In order to determine whether metapristone accumulated in blood over a long period of multiple dose administration, metapristone levels in blood were monitored at 6 h post dosing on day 1 and 10 during a consecutive 10-day oral dosing period with metapristone at 45 mg/kg/day. There was significantly different in *C*_*max*_ of metapristone between day 1 and day 10 in male and female groups (Fig. 3A). Repeated administration of metapristone over the 10 day period resulted in about 6-fold accumulation of metapristone in blood in comparison with that on day 1, and the accumulation in females was more significant than in males.

### Tissue distribution of metapristone

Metapristone in various tissues of rats at different time points was shown in Fig. 3B. At 6 h after oral administration, metapristone was widely distributed into various tissues, and the highest level of metapristone was found in the liver, the next ranked tissue was the lungs followed by kidney and ovary. We also detected metapristone in rat brain. Metapristone in liver (222.45 ± 23 µg/g) was higher than in other tissues. In general, the tissue levels of metapristone were higher than in whole blood at the same time points (Fig. 3B).

### Metabolism rate of metapristone in liver microsomes

**Figure 4:**
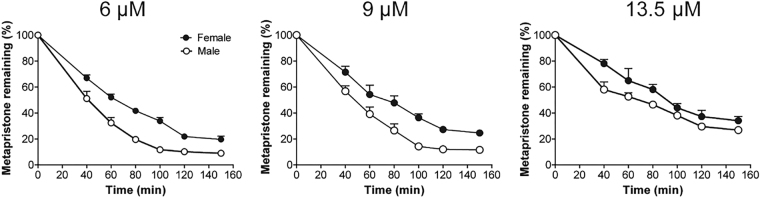
Metabolism rate of metapristone when incubated with male or female rat liver microsomes. Metapristone was added to the liver microsomal mixtures of male or female rats at the final concentrations of 6, 9 and 13.5 µM at 37 °C, followed by quantitative analysis of the remaining metapristone.

Metabolism rate of metapristone was determined at six time points between 40 and 150 min in both sexes of rat liver microsomes. The rat liver microsomes were prepared as we previously described^8^. Figure 4 showed the metabolic stability of metapristone in both sexes of rats. Incubation of 6, 9 and 13.5 µM metapristone at 37 °C with rat liver microsomes for 1 h resulted in 47.4–67.6% and 35.1–48.9% degradation of metapristone in male and female rat liver microsomes, respectively. At 2 h, only 10.2, 14.2 and 29.6% of the starting 6, 9 and 13.5 µM of metapristone remained in male rat liver microsomes; whereas, there were 21.9, 27.2 and 37.3% of the starting 6, 9 and 13.5 µM metapristone remained in female rat liver microsomes. The result indicated that 1) metapristone was metabolized relatively slow by rat liver microsomes in comparison with other drugs that we studied previously^9,10^; 2) metapristone had slower metabolic rate in female rat liver microsomes than in male ones.

### CYP1A2 and 3A4 are responsible for metapristone metabolism

To determine whether metapristone is a CYP450 substrate and, if so, which CYP isoform(s) are involved in its biotransformation, we conducted reaction phenotyping to determine whether the CYP isoform is polymorphically expressed, or is highly inducible. First, to achieve results that are considered good practices, we carried out the quality control study by using control incubations that excluded substrate metapristone, NADPH, or microsomes, respectively, from the control incubations to rule out the potential interfering factors from the microsomal study. No significant metabolites of metapristone presented in the control incubations that contained the untransfected cells, or excluded NADPH. After the quality control study, we could surely subtract the interfering background noises from metapristone reaction signals, and present reliable data based on chromatographic peak areas to verify the phenotyping results.

**Figure 5:**
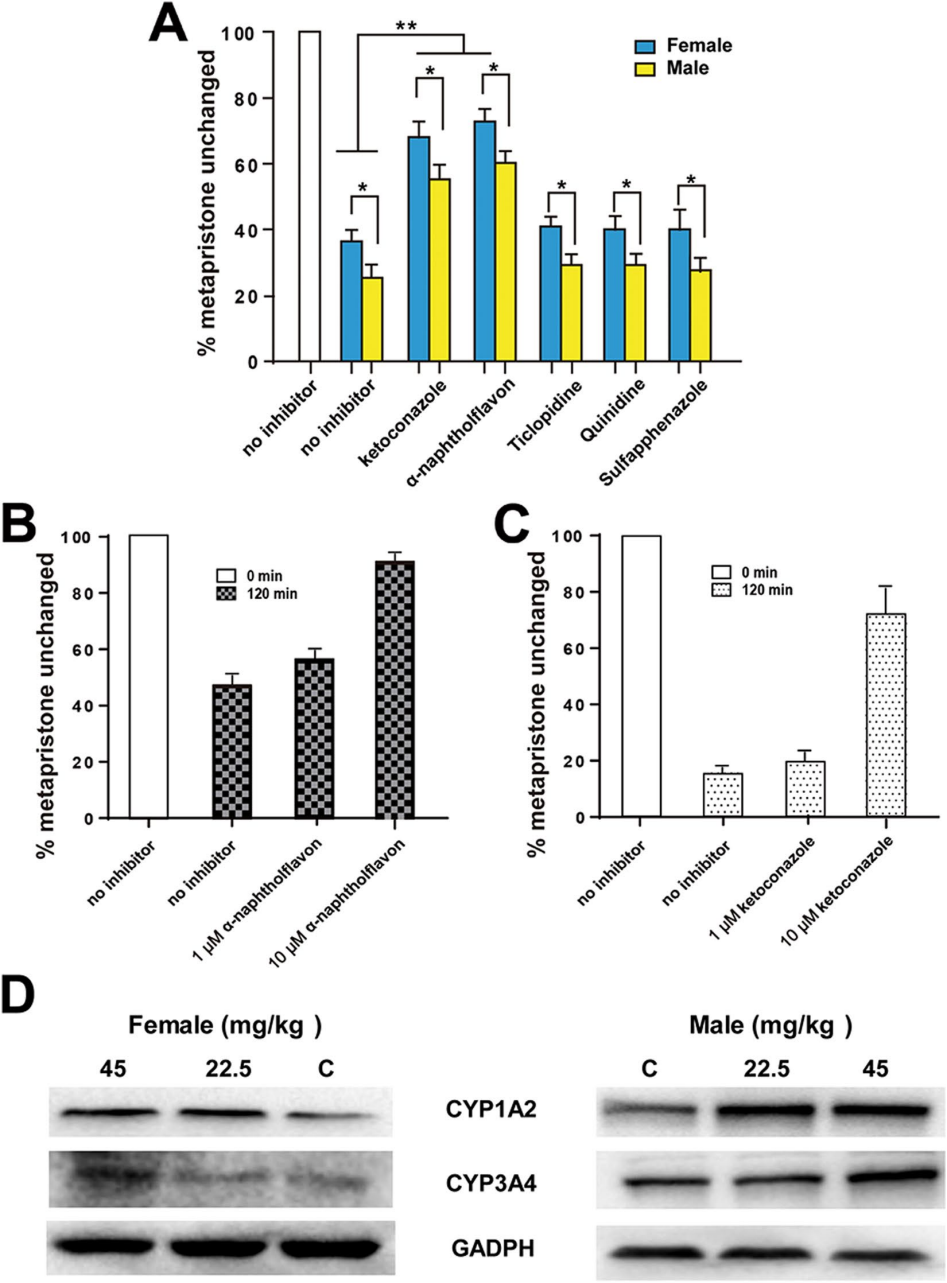
Changes of metapristone metabolism rate induced by male and female liver microsomes in the presence and absence of CYP inhibitors. (**A**) No significant differences in metabolism rate of metapristone (9 µM) between controls and CYP450 inhibitor sulfaphenazole (CYP2C9), quinidine (CYP2D6), or ticlopidine (CYP2C19); whereas CYP450 inhibitor ketoconazole (CYP1A2) and naphthoflavone (CYP3A4) significantly prevented metapristone from degradation in comparison with the controls during the 120 min incubation. The data also show statistically significant differences in metapristone metabolism between female and male liver microsomes in the presence of the five inhibitors. (**B**) α-naphthoflavone produced concentration-dependent prevention against metapristone metabolism induced by human CYP3A4; (**C**) ketoconazole produced concentration-dependent prevention against metapristone metabolism induced by human CYP1A2. (**D**) up-regulated activities of rat liver CYP1A2 and CYP3A4 after 7-day administration of metapristone. Data represent the mean ± s.d. (n = 3); *P < 0.05; **P < 0.01 of statistically significant difference (Student *t* test).

We then incubated metapristone with male or female liver microsomes in the presence of one of five specific CYP450 isoform inhibitors at the same molar concentrations to determine which inhibitor could specifically antagonize metabolism of metapristone, and identify the individual CYP450 isoforms involved in metapristone’s sex-specific metabolism. Incubations of metapristone (9 µM) with male or female rat liver microsomes in the presence of the five inhibitors resulted in changes in metapristone levels by sulfaphenazole, quinidine, and ticlopidine to the control level (Fig. 5), indicating that CYP450 2C9, 2D6, and 2C19 might not be the primary CYP450 isoforms that participate in the metabolism of metapristone. Whereas, ketoconazole and α-naphthoflavone significantly prevented metapristone from degradation by the liver microsomes as shown by the decrease in the degradation rate of metapristone when it was co-incubated with ketoconazole or α-naphthoflavone in the liver microsomal mixture (Fig. 5B,C). In addition, this experiment demonstrated the statistically significant differences in metapristone metabolism between female and male liver microsomes in the presence of the five inhibitors (Fig. 5A).

We then used cDNA-expressed recombinant human CYP1A2 and 3A4 and their specific inhibitors to further confirm results obtained from the above microsomal study. When α-naphthoflavone or ketoconazole was incubated with 9 µM metapristone in the presence of CYP1A2 or CYP3A4 for 120 min, the two specific inhibitors reversed CYP1A2- or CYP3A4-induced metabolism of metapristone in a dose-dependent manner (Fig. 5B,C). For example, in the absence of the CYP1A2 inhibitor α-naphthoflavone, 47.9% of metapristone remained after 120 min incubation with CYP1A2. Addition of α-naphthoflavone (10 µM) to the microsomal incubation prevented the CYP1A2-induced metabolism of metapristone and made 89.9% of metapristone survived after 120 min incubation. Similarly, in the absence of CYP3A4 inhibitor ketoconazole, there were 16.3% of metapristone remaining after 120 min incubation with CYP3A4. Addition of ketoconazole (10 µM) to the microsomal incubation prevented the CYP3A4-induced metabolism of metapristone and resulted in 72.9% metapristone unchanged after 120 min incubation.

To further confirm CYP1A2 and CYP3A4 are the primary CYP isoforms that metabolize metapristone, we administered oral metapristone (22.5 and 45 mg/kg/day for 7 days) to male and female rats, respectively, and measured the activity of liver CYP1A2 and CYP3A4 by using the western blotting assay. We selected western blot staining density from the controls as the basis (100%) to compare changes in CYP1A2 and CYP3A4 activity after metapristone treatment. As shown in Fig. 5D, female CYP1A2 was up-regulated to 120 ± 16% and 147 ± 21%, and male CYP1A2 was up-regulated to 156 ± 19% and 163 ± 12%, respectively, by metapristone at 22.5 and 45 mg/kg/day. Regarding the CYP3A4 activity, female CYP3A4 was up-regulated to 107 ± 13% and 128 ± 12%, and male CYP3A4 was up-regulated to 115 ± 14% and 153 ± 17%, respectively, by 22.5 and 45 mg/kg/day of metapristone. Metapristone at 45 mg/kg showed significant up-regulation of both CYP1A2 and CYP3A4 activities of both sexes in comparison with the controls (*P* < 0.05) with male CYP1A2 and CYP3A4 activities being up-regulated more significantly than the female counterparts (*P* < 0.05).

## Discussion

This is the first report that not only characterized sex-related pharmacokinetics of metapristone, but also analyzed the related mechanisms at the molecular and cellular levels. In general, metapristone exhibited blood concentrations higher in female rats and dogs than in males as demonstrated by both a single dose (Fig. 1) and repeated dose administration (Fig. 3A). The differences reflected on many concentration-related pharmacokinetic parameters (Tables 1 and 2). These differences might result from our findings that female rat liver microsomes and the related CYP1A2 and 3A4 metabolized metapristone much slower than their sex partners (Figs 4 and 5).

The doses designed in the present study for rats and dogs are based on the interspecies equivalent surface area dosage conversion factor *Km* in order to make the interspecies pharmacokinetic parameters comparable between species (including human) based on their metabolism rate per body surface area^10–12^. For instance, in rats, the dose of 22.5 mg/kg is equivalent to a dose of 132 mg/m^2^ based on *Km* 5.9 kg/m^2^ ( = 22.5 mg/kg × 5.9 kg/m^2^); in dogs, 5.6 mg/kg is equivalent to 112 mg/m^2^ based on *Km* 20 kg/m^2^ ( = 5.6 mg/kg × 20 kg/m^2^). The study showed that in general, metapristone has higher blood levels in rats than in dogs, assuming dose equivalency to both rats and dogs on the basis of mg/m^2^. Although we found that the sex-related pharmacokinetic differences of metapristone between female and male dogs are statistically significant (*P* < 0.05; Table 2), the differences are less profound than those found in rats. There are some physiological factors that could cause the species differences in pharmacokinetics as we and Lin reported^10,13^, including, but not limited to, 1) blood flow: dogs have a higher hepatic blood flow than mice and rats, and the hepatic blood flow regulates a drug’s clearance^10^; 2) drug hepatic metabolism: there are interspecies differences in CYP450 activities as we and Lin reported^10,12,13^. The differences could produce the species differences in pharmacokinetics; 3) other factors such as the difference in the urinary excretion via renal distribution processes, plasma protein binding.

The non-compartmental pharmacokinetic analysis for a single-dose metapristone showed significant sex-specific differences (*P* < 0.05) in pharmacokinetic parameters, including *C*_*max*_, *CL/F*, *V*_*d*_ */F*, *F* and *AUC*(_*0−t*)_ at three dose levels in both rats and dogs. As the general rule-of-thumb, drug blood concentrations are dependent on the volume of distribution, *CL*, and drug’s intrinsic properties. Some drugs show large *V*_*d*_. For example, Rolofylline has *V*_*d*_ 239 L^14^; Solithromycin has *V*_*d*_ > 500 L^15^ and Chloroquine has *V*_*d*_ 15,000 L. Lipophilic molecules usually have a large *V*_*d*_ because they easily sequester into fat tissue. Metapristone showed the relatively higher volume of distribution and slower *CL* than those drugs we studied before^8,9,16–20^, which make its residence time in body long (Tables 1 and 2). Although methods used for predicting a drug’s *V*_*d*_ are based on the experimentally-determined physicochemical parameters of the tested drug, which can predict the *V*_*d*_ close to its actual value, one must keep in mind that drug- or plasma-based models refer to the process of drug penetration into hypothetically homogeneous compartments as ‘tissue penetration’. This concept may be misleading, as it does not take into account of the uniqueness of separately heterogeneous organ systems^21^. Therefore, it rarely corresponds to a real volume, such as plasma volume, extracellular water or total body water. Drug distribution may be to any one or a combination of the tissues and fluids of the body. Furthermore, binding to tissue components may be so great that the *V*_*d*_ is many times of the total body size^10^. Most importantly, one must consider the interstitial space fluid as the actual space for a drug.

We showed that mifepristone possesses the unique property of enterohepatic circulation^22^, and high plasma protein binding rate at 90%^23^. The enterohepatic recycling could prolong the half-life of the recycled drug like metapristone. These two properties of metapristone plus its *V*_*d*_ */F* and *CL* together make metapristone *T*_*1/2*_ and *MRT* longer than those drugs that we studies. Because of its long *T*_*1/2*_ and *MRT*, multiple administration of metapristone for 10 days produced cumulative blood concentrations of metapristone higher than those on day 1, and the cumulated levels were significantly higher in females than in males (Fig. 3A), confirming again the sex-related differences in pharmacokinetics of metapristone.

Tissue distribution of metapristone following its single dose administration revealed that metapristone was primarily disposed in liver followed by spleen, kidney, ovary, lung and heart (Fig. 3B). In our previous studies, we reported the reversible liver toxicity caused by 30-day oral administration of mifepristone and metapristone to rats at the high dose of 200 mg/kg/day^6^. Some drugs dispose higher in liver^10^, but others do not^20^. The high disposition of metapristone in livers found in the present study further assists us in understanding the reason why the high dose and long-term administration of metapristone and mifepristone could produce liver toxicity.

Using liver microsomal system as we previously reported^10,11^, we found that female rat liver microsomes metabolized metapristone much slower than male liver microsomes did (Fig. 4), and the result was consistent with the result obtained from P450 reaction phenotyping study using recombinant cDNA-expressed human CYPs in conjunction with their specific CYP inhibitors, in which, we found that 1) in the presence and absence of specific CYP inhibitors, female rat liver microsomes metabolized metapristone slower than male rat liver microsomes; 2) CYP1A2 and CYP3A4 inhibitors produced dose-dependent prevention of metapristone metabolism; and 3) activities of both CYP1A2 and CYP3A4 are higher in males than in females (Fig. 5). These results suggest that CYP1A2 and CYP3A4 are the predominant CYPs involved in metapristone metabolism.

**Figure 6:**
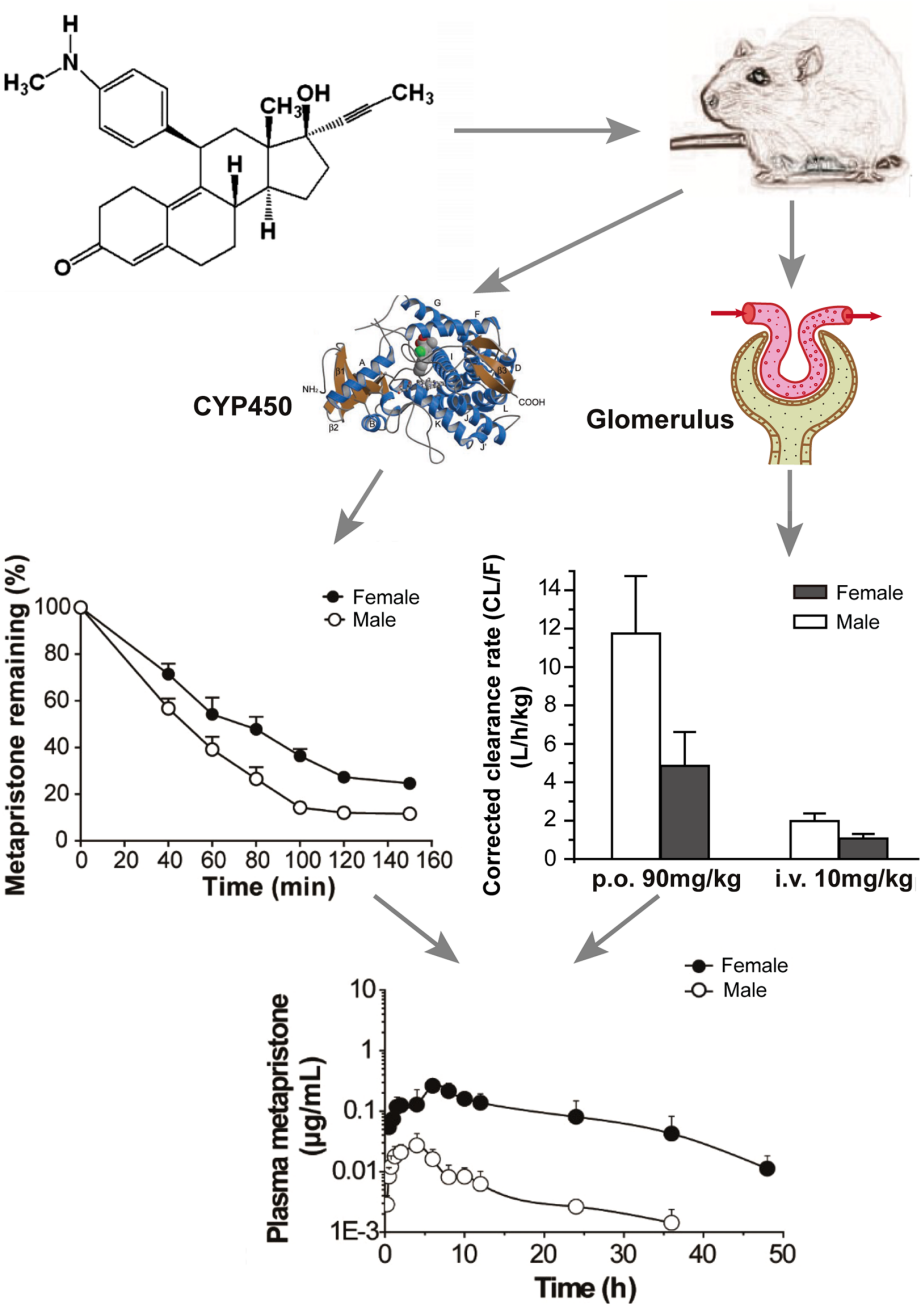
Conclusive schematic of the sex-related pharmacokinetics of metapristone and the related mechanisms. Rat blood samples were obtained after metapristone administration, and analyzed by using the validated LC/MS/MS method. The analysis revealed that metapristone metabolized by liver microsomes was slower in females than in males probably because of lower activities of CYP1A2 and CYP3A4 in females (left panel). The corrected clearance (*CL/F*, representing the renal clearance) of metapristone (p.o. and i.v.) from the body was significantly slower in females than in males (right panel; the data were extracted from Table 1). As a result, plasma concentrations of metapristone in female rats were higher in males.

Sex-dependent pharmacokinetics could result in significant sex-related differences in the drug’s efficacy and toxicity^24,25^. Sex-related drug metabolism constitutes one of the major reasons for sex-based pharmacokinetics and toxicity^26–31^. However, our study provides more systematic analysis to address the sex-related differences in metapristone pharmacokinetics using sex-specific rodent and non-rodent, sex-specific rat liver microsomes and CYP isomers (Fig. 6). The study demonstrated that metapristone had sex-related pharmacokinetics. It disposed more to the CYPs-abundant tissues such as liver and intestine. The sex-related differential expression of CYP450 1A2 and 3A4 was responsible for the sexual dimorphic metabolism of metapristone, resulting in its sex-related pharmacokinetics in animals.

In conclusion, the present study revealed, for the first time, the sex-related pharmacokinetics of the metapristone, and demonstrated that females metabolized metapristone slower than males, resulting in the corresponding high blood and tissue concentrations. The liver microsomal CYP1A2 and CYP3A4 are primarily responsible for the sex-related pharmacokinetics. Our future studies will investigate the effects of sex hormones on the sex-related pharmacokinetics of metapristone *in vitro* and *in vivo*.

## Methods and Materials

### Chemicals and reagents

Mifepristone (purity > 98.0%) was purchased from New Hua Lian Pharmaceutical Corporation (Shanghai, China). Metapristone was synthesized from mifepristone as we reported previously with minor modification^1^. The purity of metapristone was determined > 98.0%. Levonorgestrel (internal standard, IS, 98.0% purity) was provided by National Institutes for Food and Drug Control (Beijing, China).Rat liver microsomes used in this study were purchased from the Research Institute for Liver Disease Co (Shanghai, China). The NADPH regenerating system was purchased from Sigma Aldrich (USA). HPLC-grade methanol and formic acid were purchased from Merck Drugs & Biotechnology Company. Analytical grade ethyl acetate was provided by Shanghai chemical factory (Shanghai, China). HPLC-grade water was obtained from a Milli-Q water purification system (Millipore, Bedford, MA, USA). All other chemicals and reagents used were of analytical grade. Drug-free whole blood was collected from adult and healthy male and female rats and serum was separated separately at 4000 g for 10 min and stored at −80 °C until use.

### Animals

Sprague Dawley (SD) rats (180–220 g, males and females) were obtained from the Shanghai Laboratory Animal Center (Shanghai, China). They were housed in a separate room, and caged according to sex and dose levels. All animals were housed in specific pathogen-free conditions. They were kept at standard conditions of temperature, humidity, and received water and food *ad libitum* and allowed to acclimatize in the laboratory for at least 1 week before experiment. Before administration, the animals were fasted overnight with free access of water. Beagle dogs (7.5–8.9 kg, males and females) were obtained from the Laboratory Animal Center (Fuzhou, China). All studies involving animals were carried out in accordance with the NSFC regulation concerning the care and use of experimental animals and approved by the Institutional Animal Care and Use Committee of Fuzhou University (#13/2014) to reduce the suffering and use of animals.

### Liquid chromatographic and mass spectrometric conditions

The chromatographic separation of metapristone and IS was performed on an ACQUITY UPLC system using the ACQUITY UPLC BEH C_18_ column (1.7 µm, 50 mm × 2.1 mm, id, Waters, USA) at 35 °C and a flow rate of 0.3 mL/min, as we described previously^7^. The mobile phase was composed of methanol (A) and aqueous 0.1% (v/v) formic acid (B) according to the following gradient program (T min/% A): 0/40, 1/95, 2.5/95, 2.8/40, 6/40. The injection volume was 5 µL. After each injection, a needle wash process was conducted for a sequential wash with methanol–water solution (50:50, v: v) and methanol–water solution (20:80, v-v).The Quattro Micro mass Spectrometer (Waters Corp., Milford, MA, USA) was equipped with an electrospray ionization (ESI) source under the following operating parameters: capillary voltage 3.2 KV, cone voltage 40 V, source temperature 110 °C, desolvation temperature 500 °C, desolvation gas (nitrogen) 800 L/h, cone gas (nitrogen) 50 L/h. Argon was used as the collision gas at a pressure of approximately 0.15 mPa. The entrance and exit energies of the collision cell were set at −1 and 2 V, respectively. Quantitative analysis was performed using MRM method in a positive mode: metapristone at *m/z* 416.3 → 119.9 with collision energy set to 40 V and IS at *m/z* 313.1 → 109 with collision energy set to 35 V, with a scan time of 0.20 s per transition. The dwell time for metapristone and IS was set to 0.15 s. All data was acquired in centroid mode and processed using Masslynx 4.1 Software (Waters Corp., Milford, MA, USA).

### Pharmacokinetic study with metapristone

For pharmacokinetic study, rats were fasted overnight with free access to water using procedures reported previously^7^. Ninety-six SD rats (forty-eight males and forty-eight females) were randomly divided into eight single-dose groups. Rats (six animals/sex/dose) were given a single oral dose of 22.5, 45 and 90 mg/kg metapristone (in soybean oil solution) by intragastric administration or 10 mg/kg metapristone by intravenous bolus injection. Rat blood samples (0.25 mL) were collected from the ocular vein into heparinized tubes prior to and at 0 (before administration), 0.5, 1.0, 1.5, 2.0, 4.0, 6.0, 8.0, 12, 24, 36 and 48 h post-dosing in female rats or 0.5, 1.0, 1.5, 2.0, 4.0, 6.0, 8.0, 12, 24, 30 and 36 h post-dosing in male rats. In the repeated dose study twenty-four rats (six animals/sex/dose) received the same dosing regimen at 45 mg/kg metapristone for 10 days. Blood samples were collected after oral administration on day 1 and 10 at *T*_*max*_. The samples were stored at −80 °C until analysis. Beagle dogs (three animals/sex/dose) were dosed by gavage at 5.62, 11.25, 22.5 mg/kg metapristone (in soybean oil solution). Dog blood (0.25 mL) was withdrawn from the jugular vein at 0.5, 1.0, 2.0, 4.0, 6.0, 8.0, 10, 12, 24 and 48 h after a single oral administration.

The plasma samples obtained by centrifugation at 4000 g for 10 min and stored at −80 °Cuntil analysis. To each 100 µL of plasma sample, 50 µL of internal standard solution were added. Metapristone was then separated and analyzed by the LC/MS/MS method according to the previously described procedures^18^. Peak area ratios of metapristone to the internal standard were plotted against theoretical concentrations. The maximum plasma concentration (*C*_*max*_), the time to reach *C*_*max*_ (*T*_*max*_) were determined directly from experimental data. The other pharmacokinetic parameters such as elimination half-life (*t*_*1/2*_), and mean residence time (*MRT*) were calculated by using the non-compartmental pharmacokinetics data analysis software of DAS version 3.2 (Bio Guider Co., Shanghai, China). The area under the plasma concentration-time curve from zero to infinity was calculated by using the linear trapezoidal rule with extrapolation to infinity with terminal constant eliminate rate (*k*_*e*_). Bioavailability was calculated as (AUC_p.o._/AUC_i.v._) x (dose_i.v._/ dose_p.o._) 100% as we previously described^12^.

### Tissue distribution study with metapristone

Sixteen SD rats (four females/group) were dosed by gavage with 45 mg/kg of metapristone to carry out tissue distribution study, as we previously described^10^. Rats were sacrificed at 6 h and 24 h after dosing in order to collect blood, heart, liver, spleen, lung, kidney, brain, intestine and ovary for quantitative analysis of metapristone concentrations. Tissue samples were immediately washed in normal saline to remove the blood or content, and blotted dry with filter paper. Tissue samples (0.2 g) were individually homogenized with normal saline (0.8 mL). Tissue homogenates (100 µL) were processed as the plasma samples. The detailed method validation for analysis of metapristone in plasma, tissue homogenates, and liver microsomal samples is provided in the Supplementary materials.

### *In vitro* microsomal metabolism of metapristone

The microsomal assay was similar to that described previously^11^. Briefly, metapristone (6, 9, 13.5 µM) was incubated with male and female at liver microsomes, respectively, in an NADPH-generating system containing 1 mM NADP, 10 mM glucose-6-phosphate, 1 U/mL glucose-6-phosphate dehydrogenase and 4 mM MgCl_2_ in 100 mM potassium phosphate buffer (pH 7.4). Reaction mixtures were prepared in duplicate and were pre-incubated for 5 min at 37 °C. The actions were then initiated by the addition of NADPH solution. The final volume of each reaction mixture was 200 µL. Negative control reactions were prepared by incubating mixtures that excluded metapristone from the mixture. For negative control incubation where microsomes were excluded, they were added back to the reaction mixture after quenching with methanol. Samples were removed at 0, 40, 60, 80,100, 120 and 150 min, and vortex-mixed with cold ethyl acetate to stop the reaction. After centrifugation, a portion of each resulting supernatant was analyzed by mass spectrometry for unchanged metapristone.

### Sex-related isoforms of CYP450 study with metapristone

To identify CYP450 isomers which were responsible for the sex different metabolism of metapristone, both CYP450 inhibitors and cDNA-expressed recombinant human CYPs were used^11^. Male or female at liver microsomes were incubated with metapristone (9 µM), NADPH-generating system and the well-demonstrated CYP inhibitors: quinidine at 10 µM (CYP2D6), ticlopidine at 10 µM (CYP2C19), α-naphthoflavone at 10 µM (CYP1A2), sulfaphenazole at 10 µM (CYP2C9), ketoconazole at 10 µM (CYP3A4)^32^. Different selective CYP inhibitors were dissolved in methanol prior to addition to the incubation mixtures, and the resulting solutions were incubated at 37 °C for 120 min in male and female at liver microsomes. The reactions of the control groups with control rat liver microsomes (heated to 80 °C for 5 min to inactivate enzymes) to inactivate enzymes or without NADPH were also included. The no inhibition group contained chemical inhibitors but the same amount of methanol (0.1%). The cDNA-expressed recombinant human CYPs were prepared from insect cells transfected with cDNAs encoding for human CYP1A2 or CYP3A4. Reaction mixtures containing human cDNA expressed CYP1A2 or CYP3A4, the above-mentioned NADPH-generating system, selective CYP inhibitors and 100 mM potassium phosphate buffer (pH 7.4) were pre-incubated for at 37 °C for 120 min. Each reaction was then started by the addition of metapristone (9 µM) with subsequent mixing of each sample by inversion. The samples were immediately removed and mixed with cold ethyl acetate to stop reaction at 0 and 120 min of incubation. Metapristone were identified by the LC/MS/MS method. Peak areas formed were used for quantitative analyses. Control incubation mixtures included mixtures without inhibitors, and mixtures with untransfected insect cell microsomes used as microsomal control, and mixtures that contained methanol instead of inhibitor (methanol control). Quantitative analyses were performed by comparing the peak areas of the inhibition reactions to their respective methanol controls. The total organic solvent content of the *in vitro* reaction mixtures was less than 2%.

### Western Blotting for measuring CYP1A2 and 3A4

The total microsomal protein was extracted from rat livers prepared by diluting liver microsomal suspension with 1 volume of immunoprecipitation assay lysis buffer (150 mM NaCl, 1% NP-40, 0.5% sodium deoxy-cholic acid, 0.1% SDS, and 50 mM Tris-Cl, pH 7.5) for 15 min and then centrifuged at 13,000 rpm 4 °C for 10 min. Aliquots containing 20 µg of protein were separated by SDS-PAGE on a 10% gradient gel (Invitrogen, USA) and transferred to a PVDF membrane that was blocked with 5% non-fat dry milk in Tris-buffered saline with Tween 20 (200 mM Tris–HCl, 1.37 M NaCl, 0.1% Tween 20, pH 7.6) for 1 h. The membrane was then incubated with rabbit anti-rat CYP1A2 polyclonal antibody (1:2000 dilution), rabbit anti-rat CYP3A4 polyclonal antibody (1:1000 dilution) and glyceraldehyde-3-phosphate dehydrogenase (GAPDH) antibody (1:2000 dilution), overnight at 4 °C. After rinsing five times with buffer TBST at room temperature, the membrane was incubated with horseradish peroxidase (HRP)-labeled anti-rabbit immunoglobulin G (IgG) anti-body (Santa Cruz Biotechnology, USA). The immunoreactive bands were visualized using an enhanced chemiluminescence detection system (ChemiDoc™XRS + , Bio-Rad, Hercules, CA, USA) and quantified by using densitometry Image J 1.41 software.

### Statistical analysis

Data are expressed as the mean ± s.d. Statistical analysis was performed by an analysis of variance (ANOVA) with α = 0.05 as the minimal level of significance.

## Electronic supplementary material

Dataset
