# Supplementary material for: Sex-related pharmacokinetic differences and mechanisms of metapristone (RU486 metabolite)
Source: Sci Rep. 2017 Dec 7;7:17190. doi: 10.1038/s41598-017-17225-0 (PMC5719405; doi:10.1038/s41598-017-17225-0)
Supplement: Supplementary file 1 — Dataset [file 41598_2017_17225_MOESM1_ESM.doc]

**Supplementary**

**Sex-related pharmacokinetic differences and mechanisms of metapristone (RU486 metabolite)**

Wenge Chen^1^, Yingying Xiao^1^, Jianzhong Chen, Jian Liu, Jingwei Shao, Tao Li, Yewei Zhu, Ji Ma, Yu Gao, Jichuang Wang, Jianguo Xu, Yusheng Lu, Lee Jia*

Cancer Metastasis Alert and Prevention Center, and Fujian Provincial Key Laboratory of Cancer Metastasis Chemoprevention, Fuzhou University, Fuzhou 350002, China.

**Liquid chromatographic and mass spectrometric conditions**

The chromatographic separation was performed on an ACQUITY UPLC system as described in the text.

**Preparation for stock and working solutions**

Stock standard solutions of metapristone and internal standard (IS) were prepared by dissolving proper amount of accurately weighted reference compounds in 90% methanol solution, respectively. Both stock standard solutions were then serially diluted with 50% methanol solution to provide eight standard working solutions for metapristone (14.2-5680 ng/mL) and an IS working solution (426 ng/mL), respectively. All of the solutions were stored at 4℃ and brought to room temperature before use.

**Preparation for calibration standards and quality control samples**

Calibration standards were prepared daily by spiking appropriate standard working solutions (50 µL of metapristone) to 100 µL of blank supernatants of tissue homogenates. Effective concentrations were 7.1, 14.2, 28.4, 71, 355, 710, 1420, 2840 ng/mL of metapristone. The low, medium, and high quality control (QC) samples were prepared in a similar way as the calibration standards to give three final concentrations of 14.2, 355, 2840 ng/mL for metapristone. The spiked samples (calibration standards and QC samples) were then treated with the following liquid-liquid extraction (LLE) procedure as described below.

**Tissue and microsomal sample preparation**

To an aliquot of 100 µL of samples, 50 µL of IS solution (426 ng/mL) and 50 µL of 50% methanol solution were added to a 1.5 mL polypropylene micro-centrifuge tube. The mixed sample was then extracted with 1.0 mL of [ethyl](app:ds:ethyl) [acetate](app:ds:acetate) by vortex-mixing for 5 min. After centrifugation at 2,348 × g for 10 min, the upper organic layer (950 µL) was carefully removed, and transferred to another clean test tube and evaporated to dryness under nitrogen at 45℃. The dried residue was reconstituted in 100 µL of methanol-water solution (50:50, v/v) followed by vortex-mixing for 1 min. After centrifugation at 22,000 × g for 15 min, a 5 µL aliquot of the supernatant was injected into the chromatographic systems for analysis.

**Method validation of tissue and microsomal samples**

The calibration curve of metapristone was constructed using spiked tissue homogenates or microsomal samples at eight levels in the range of 7.1-2840 ng/mL with weighted (1/x^2^) least square linear regression method through measurement of the peak area ratio of metapristone to IS. The LLOQ was defined as the lowest concentration on the calibration curve, at which an acceptable precision (expressed as relative standard deviation, RSD, %) should be within 20% and accuracy (expressed as relative error, RE, %) within ± 20%. The LLOQ was evaluated by analyzing spiked samples prepared in four replicates.

To evaluate the precision and accuracy of this assay, QC samples at three levels were analyzed in six replicates on the same day and on three consecutive days, respectively. Each run consisted of two calibration curves and six replicates of every concentration. The assay accuracy was expressed by RE and precision by RSD.

The extraction recovery of metapristone at three QC levels were determined by comparing the peak areas of metapristone in the samples that were spiked with metapristone prior to extraction of blank samples with those in the samples to which metapristone had been added post-extraction of blank samples. The matrix effect at three QC levels was measured by comparing the peak response of samples spiked post-extraction (A) with that of pure standard solution containing equivalent amount of metapristone (B). The ratio (A/B × 100%) was used to evaluate the matrix effect. The extraction recovery and matrix effect of IS were also simultaneously evaluated using the same method.

The stability of metapristone in liver microsomes was assessed by analyzing six replicates of low, medium and high QC samples during the sample storage and processing procedures. The freeze-thaw stability was measured after three freeze-thaw cycles. Post-preparation stability was estimated by analyzing QC samples at 0 and 12 h in the refrigerator at 4℃. Six aliquots of QC samples were stored at –20 ℃ for 30 days and at ambient temperature for 4 h to determine long-term and short-term stability, respectively. All QC samples were detected by using calibration curves of freshly prepared standards.

**Specificity**

We defined the specificity as the ability to assess unequivocally the analyte in the presence of components that may interrupt the quantitative measurement. In this study, no significant interfering peaks from endogenous substances were observed at the elution times for metapristone and IS under the current optimal conditions.

**Linearity and the lower limit of quantification.**

The peak area ratios of metapristone to IS in tissue homogenates varied linearly within the concentration range from 7.1-2840 ng/mL. The best linear fit and least-square residual for the calibration curve were achieved with 1/x^2^ weighting factor. Using the metapristone concentrations as the X-abscissa, and the peak area ratio of metapristone to IS as the Y-ordinate, we established the standard curve with the regression equation Y= 3.64x + 0.0049, r^2^ = 0.9954 for the representative liver tissue homogenate samples (Fig. 1-Suppl.). The typical regression equation for liver microsomal calibration curve was Y= 0.4413x + 0.0026, r^2^ =0.9932 (Fig. 2-Suppl.). For metapristone, the present UPLC/MS/MS method could reach RE at -5.6%, and the intra- and inter-day variations at 4.5% and 10% RSD, respectively.

**Precision and accuracy.**

The assay accuracy ranged from -2.3 to 8.6% RE, and the intra- and inter-day variations ranged from 2.4 to 5.1% and 6.7 to 8.0% RSD at three QC concentrations, respectively. The present method had the same excellent intra- and inter-day precision and accuracy at three levels as those reported before. And the results showed that the method met our desired acceptance criteria of less than or equal to 10% RSD and RE.

**Extraction recovery and matrix effect.**

The extraction recoveries of metapristone from QC samples were 80.7, 81.9 and 82.3% at low, medium and high concentrations, respectively. The recovery of IS was 86.9%. With regard to the matrix effect, all the ratios defined above were within the acceptable limits (85-115%). No significant matrix effect for metapristone was observed, suggesting that ion suppression and enhancement from plasma are negligible for this method.


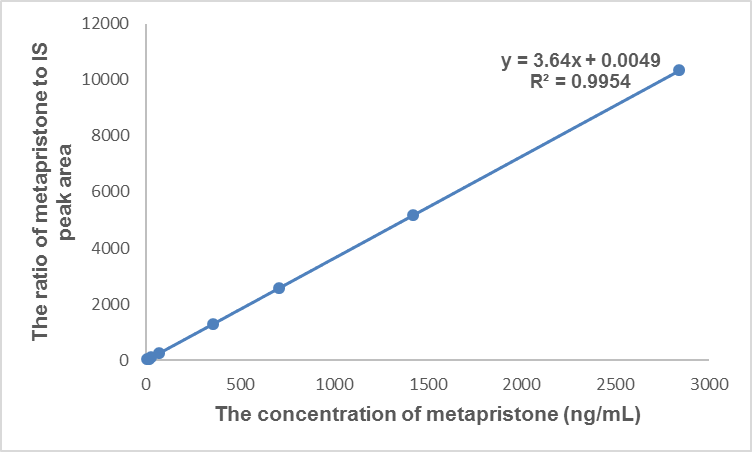


Fig.1-Suppl. Typical regression equation and calibration curve for metapristone spiked into rat liver homogenate.


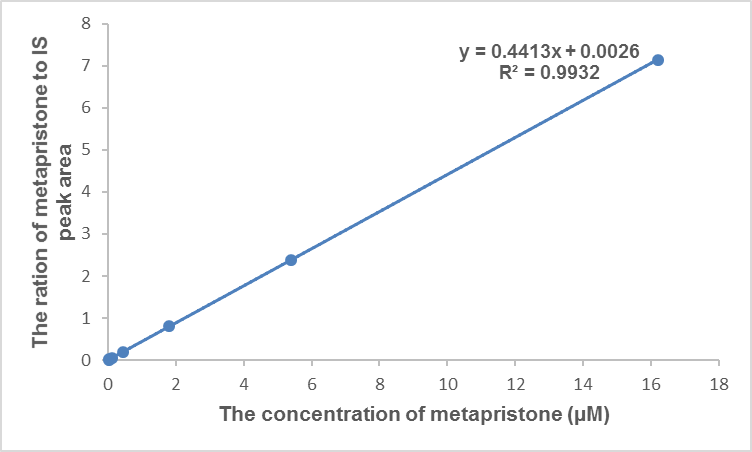


Fig. 2-Suppl. Typical regression equation and calibration curve for metapristone spiked into rat liver microsomes.
